# Field-Usable Lateral Flow Immunoassay for the Rapid Detection of White Spot Syndrome Virus (WSSV)

**DOI:** 10.1371/journal.pone.0169012

**Published:** 2017-01-03

**Authors:** Prabir Kumar Kulabhusan, Jyutika M. Rajwade, Vimal Sugumar, Gani Taju, A. S. Sahul Hameed, Kishore M. Paknikar

**Affiliations:** 1 Nanobioscience Group, Agharkar Research Institute, Pune, India; 2 OIE Reference Laboratory for WTD, C. Abdul Hakeem College, Melvisharam, Tamilnadu, India; VIT University, INDIA

## Abstract

**Background:**

White spot disease (WSD), a major threat to sustainable aquaculture worldwide, is caused by White spot syndrome virus (WSSV). The diagnosis of WSD relies heavily on molecular detection of the virus by one-step PCR. These procedures are neither field-usable nor rapid enough considering the speed at which the virus spreads. Thus, development of a rapid, reliable and field-usable diagnostic method for the detection of WSSV infection is imperative to prevent huge economic losses.

**Methods/Principal Findings:**

Here, we report on the development of a lateral flow immunoassay (LFIA) employing gold nanoparticles conjugated to a polyclonal antibody against VP28 (envelope protein of WSSV). The LFIA detected WSSV in ~20 min and showed no cross-reactivity with other shrimp viruses, viz. Monodon Baculovirus (MBV), Hepatopancreatic parvovirus (HPV) and Infectious Hypodermal and Hematopoietic Necrosis virus (IHHNV). The limit of detection (LOD) of the assay, as determined by real-time PCR, was 10^3^ copies of WSSV. In a time course infectivity experiment, ~10^4^ WSSV particles were injected in *Litopenaeus vannamei*. The LFIA could rapidly (~ 20 min) detect the virus in different tissues after 3 h (hemolymph), 6 h (gill tissue) and 12 h (head soft tissue, eye stalk, and pleopod) of infection. Based on these findings, a validation study was performed using 75 field samples collected from different geographical locations in India. The LFIA results obtained were compared with the conventional “gold standard test”, viz. one-step PCR. The analysis of results in 2x2 matrix indicated very high sensitivity (100%) and specificity (96.77%) of LFIA. Similarly, Cohen’s kappa coefficient of 0.983 suggested "very good agreement” between the developed LFIA and the conventional one-step PCR.

**Conclusion:**

The LFIA developed for the rapid detection of WSSV has an excellent potential for use in the field and could prove to be a boon to the aquaculture industry.

## Introduction

White spot disease (WSD), caused by white spot syndrome virus (WSSV), is one of the most devastating diseases in shrimp culture industry worldwide. Since the emergence of WSSV in 1992, the virus spread from China and Japan to Thailand, Indonesia and India. Later on, it spread to all other shrimp growing countries including the USA. The virus continues to have the greatest impact on shrimp culture to date [1–4]. WSSV is a rod-shaped dsDNA virus with large virions [5] and has been classified as a type species of genus *Whispovirus* within the family *Nimaviridae* [6]. The viral envelope consists of at least 35 different proteins, of which VP28 and VP26 are the most abundant proteins, accounting for approximately 60% of the envelope. Furthermore, VP28 is known to play a significant role in the infection process as an attachment protein helping the virus to enter the cytoplasm of the host cell [7].

WSSV infection results in the formation of small, circular white inclusions that are most noticeable in hypertrophied nuclei of ectodermal and mesodermal cells of the infected shrimp. In WSSV outbreaks on farms, mortality can reach up to 100% within 3 to 10 days of infection [8]. The infection can be transmitted horizontally through water and infected animals (mainly through crabs and wild shrimp or by cannibalization of moribund shrimp). All life stages of the penaeid shrimps, i.e. from egg to brooder, are potentially susceptible to the virus [9]. As no treatment measures are available, prevention of the infection becomes a key step to contain the disease. Accurate diagnosis of the virus at early stages is one of the most efficient strategies to monitor and control WSSV outbreak in shrimp farming industry.

The classical and well accepted methods of WSSV diagnosis such as observation of clinical symptoms in animals and histopathology have long been replaced by modern detection techniques such as PCR and immunological methods [10–13]. These methods are highly sensitive, specific and provide an accurate diagnosis. However, they are costly, time- consuming, require specialized equipment and skilled personnel and therefore not usable under field conditions [14]. Further, the sensitivity of the PCR technique varies considerably with the method of DNA extraction, template concentration and the size of the amplicon. Also, a positive PCR test, which detects a DNA fragment, is a poor indicator of viral viability [15]. Another conventional test employed for the detection of WSSV is ELISA but is not field-usable. On the other hand, dot blot and immuno chromatographic strips developed for the detection of WSSV [16, 17] are rapid, and can be used in the field to screen individual as well as pooled shrimp samples provided the viral loads are sufficiently high [18].

LFIAs that use metal nanoparticles for colorimetric detection of targets are simple, rapid, low-cost and convenient for field deployment. Gold nanoparticles (AuNPs) are the most commonly used visual readout detection labels in LFIAs developed for a variety of proteins, metal ions, hormones, bacteria, viruses, etc. [19–22]. Aquaculture industry uses several serodiagnostic techniques for the detection of WSSV. For example, reverse passive agglutination, which involves high density latex beads conjugated to a monoclonal antibody [23] is capable of detecting WSSV but takes 4 h for a result. Another method using monoclonal antibodies conjugated with gold has reported high background staining as a limitation [24]. A strip test incorporating a mixture of two different monoclonal antibodies, for binding and detection, respectively [17] would be an expensive proposition limiting its practical usability. So far, only one immuno-chromatographic assay (Shrimple) developed by EnBioTec Laboratories. Tokyo, Japan seems to have reached the marketplace. However, it is beset with problems such as false negative results, failure to detect WSSV during early stages of the infection [16], and poor penetration in markets other than Japan.

In the previous work carried out by the authors a method was standardized to raise high titer polyclonal anti-rVP28 antibody in rabbits [25]. Further, the said antibody was used in a dot blot assay format to detect very low levels of WSSV. A logical extension of this work could have been the development of an ELISA assay. However, considering the urgent need for a rapid and field-usable method, it was decided to focus our efforts on the development of LFIA. The possibility of sacrificing on sensitivity (as compared to ELISA) was factored in this choice. Once developed, we believe that the LFIA will be helpful to farmers and hatchery operators in maintaining healthy brood stock, ultimately producing good quality seed and forestalling heavy economic losses.

## Materials and Methods

### Ethics statement

Samples were collected as part of National Surveillance Program for Aquatic Animal Diseases (NSPAAD) project. Sub-project No. 24. The study was approved by National Fisheries Development Board (NFDB)-Indian Council of Agricultural Research (ICAR), Government of India.

The collection of samples was done as part of NSPAAD project; Sub-project No. 24. Therefore, specific permissions were not required.

The field studies involve only species cultivated in aquaculture farms/hatcheries. We hereby confirm that our field studies did not involve endangered or protected species.

The only experiment that required the approval of the Institutional Animal Ethics Committee (IAEC) pertains to raising of polyclonal antibodies in rabbits. This work was approved by the IAEC and is clearly mentioned under Materials & Methods section

### Assembly of the lateral flow immunoassay test strips

The assembly of LFIA strips comprised of three steps, viz. (a) production of polyclonal antibody against WSSV, (b) synthesis of colloidal gold nanoparticles-antibody conjugate and (c) assembly of LFIA strips in polypropylene cassettes.

Polyclonal antiserum against WSSV was raised in New Zealand white rabbits (2.5–3.0 Kg) using purified recombinant VP28 protein emulsified in Freund’s complete adjuvant. The protocol was approved by the Institutional Animal Ethics Committee, C. Abdul Hakeem College, Melvisharam, Tamilnadu, India vide approval no. 1011/c06/CPCSEA. After six weeks, antiserum was collected from the immunized animals, which were then subjected to standard purification steps such as ammonium sulfate precipitation (50%), overnight dialysis, and proteinA affinity column (Thermo Scientific, USA) chromatography to obtain IgG fractions. SDS-PAGE and ELISA were performed to determine the purity and titer of the antibody. Western blot was performed to confirm the absence of cross-reactivity with shrimp tissue. The purified anti-WSSV polyclonal antibody (pAb) thus obtained was stored at -80°C and used in subsequent experiments.

Gold nanoparticles (AuNPs) used as detection label in the LFIA were synthesized by standard citrate reduction method [26]. pAb (25 μg/mL) was added to 500 μL of AuNPs (pH 9 using 0.2 M K_2_CO_3_), and allowed to form AuNPs-pAb conjugate at ambient temperature with gentle stirring for 10 min. The AuNPs–pAb conjugate was centrifuged at 14,000 rpm for 15 min, 10 μL of 1% BSA was added to the soft pellets, which were then resuspended in 0.1 M phosphate buffered saline (PBS). The pAb-AuNPs conjugate formation was confirmed by UV-Vis spectroscopy, gel retardation assay and ELISA before storage at 4°C for further use.

The LFIA strips were assembled in polypropylene cassettes (5 cm x 1 cm) with sample and observation windows. The cassettes encased four overlaying components, viz. sample application pad, conjugate release matrix, high protein binding nitrocellulose membrane (0.8 μ) and absorbent pad. The polypropylene cassettes, all types of overlaying components and the necessary hardware/machinery (antibody printer, strip cutter and reagent dispenser) were procured from Advanced Microdevices Pvt Ltd, Ambala Cantt., India. The LFIA devices were assembled as per the manufacturer’s instructions. Briefly, the test (T) and control (C) lines of anti-WSSV pAb (1 mg/mL) and protein A (1 mg/mL), respectively were printed on nitrocellulose membrane (NC) using automated antibody printing machine. At one end of the NC membrane, the conjugate release matrix soaked with anti-WSSV pAb–AuNPs was placed. The sample pad was placed in such a way that it overlapped the conjugate release matrix. The absorbent pad was placed at the opposite end of the sample pad to collect excess reagents. After fabrication of these components assay strips were cut to a size of 4 x 0.4 cm (*l x b*) by using a programmable strip cutter and encased. The entire LFIA cassette fabrication procedure was carried out under dust-free conditions in a laminar air flow chamber.

### Standardization of lateral flow immunoassay

Standardization of the LFIA was done using purified WSSV. For this WSSV infected shrimp were collected and homogenized in a mortar and pestle. The tissue homogenate was made with NTE buffer [0.2 M NaCl, 0.02 M Tris–HCl and 0.02 M EDTA, pH 7.4]. The suspensions were centrifuged at 3000*g* for 20 min at 4°C. The supernatant was re-centrifuged at 8000*g* for 30 min at 4°C and the final supernatant was filtered through a 0.4 μm filter.

Initially, purified undiluted WSSV (50 μg/mL) and its different dilutions (1:10, 1:20, and 1:40) in PBS were checked in the assembled LFIA strips. PBS buffer without WSSV served as negative control. WSSV suspensions were pipetted (40μL) on the sample pad and allowed to flow through NC membrane to the other end. After 5 min, 100 μL of running buffer (0.1% SDS in PBS and 0.1% of tween-20) was added to the sample window. The appearance of red lines in the test and control zones indicating positive reaction and the time required were recorded. Suspensions of Infectious hypodermal and hematopoietic necrosis virus (IHHNV), Monodon baculo virus (MBV), and Hepatopancreatic parvovirus (HPV) were also tested in parallel to check the specificity of the LFIA.

The sensitivity of LFIA strips was determined by testing the samples by LFIA and one-step PCR simultaneously. For this purpose, double dilutions of gill tissue homogenate of WSSV infected shrimp were prepared using PBS (pH 7.4). The concentration of the protein lysate collected from WSSV infected tissue was to 100, 50, 25, 12.5, 6.25 and 3.125 μg/mL. For LFIA, 40μL of each concentration was applied to the assay strip. For one-step PCR, DNA was isolated from the above protein lysates (1 mL) and amplified using commercial PCR kit for WSSV supplied by Poseidon Biotech, Chennai, India [27].

For determining the LOD, total DNA was extracted from WSSV infected gill samples using DNA extraction buffer (Poseidon Biotech, Chennai, India) according to the manufacturer’s instructions. The viral copy number was estimated by StepOnePlus Real-Time PCR System (Applied Biosystems, USA) using TaqMan assay. Forward primer 5' CCC ACA CAG ACA ATA TCG AGA C 3' and reverse primer 5' TCG CTG TCA AAG GAC ACA TC3 ' were used to amplify a 109 bp fragment from the VP28 gene of WSSV. The TaqMan probe was labeled with 5′-5-carboxyfluorescein (FAM) and 3′-N,N,N,N-tetramethyl-6-carboxyrhodamine (TAMRA). A standard curve obtained using serial dilutions of plasmid pVP28 (full-length ORF of VP28 gene of WSSV was cloned into a pRSETB vector) was used to quantify the WSSV copy number in the gill tissue homogenate. Subsequently, the WSSV infected gill tissue samples were diluted so as to achieve 10^5^, 10^4^, 10^3^, 10^2^, 10^1^ copies. After 40 cycles the mean C_T_ values obtained were plotted against the WSSV copy number. These samples were simultaneously applied in the LFIA. The control lines (C) and test line (T) were examined visually.

### Time course infectivity experiments

WSSV (300 μg of total protein per animal, corresponding to ~ 10^4^ virus particles isolated from infected animals) was injected intramuscularly into the second abdominal segment of *Litopenaeus vannamei* [28]. Control animals were injected with the hemolymph from healthy (uninfected) shrimp. Two sets each of experimental and control animals (n = 6 per group) were maintained in 50 L tanks. The salinity of the water was maintained in the range of 5–10 ppt. The experimental animals were sacrificed at 3, 6, 12, 24, 36 and 48 h post-infection (p.i) and hemolymph, gill, head soft tissue, eye stalk, and pleopod were collected for the detection of WSSV using developed LFIA as well as one-step PCR. Each tissue was homogenized using PBS and the protein concentration was estimated using Lowry method. The samples were diluted appropriately so as to achieve a concentration of 180 μg/mL. Of these 40 μL was applied to LFIA and the DNA isolated from the tissue sample was subjected to one step PCR.

### Field level validation of lateral flow immunoassay

The LFIA was validated using 75 animal samples suspected of WSSV infection and collected from different farms located in six states of India under the National Surveillance Program for Aquatic Animal Diseases. The same samples were also subjected to one-step PCR analysis, which was used as “gold standard” for comparison**.** Based on the results obtained, the samples were classified as “true positive” (TP), “true negative” (TN), “false positive” (FP), or “false negative” (FN). The diagnostic sensitivity and specificity of the LFIA were calculated as follows:

$$Sensitivity\left(\%\right)=\frac{TP}{\left(TP+FN\right)}x100$$

$$Specificity\left(\%\right)=\frac{TN}{\left(TN+FP\right)}x100$$

Cohen’s kappa coefficient was calculated using the following formula

$$Cohen^{\prime }s\;kappa=\frac{\text{Pr}\left(a\right)-\text{Pr}\left(\text{e}\right)}{1-\text{Pr}\left(\text{e}\right)}$$

Where, Pr(a) is the relative observed agreement among raters, and Pr(e) is the theoretical probability of chance agreement using the observed data to calculate the probabilities of each observer randomly saying “yes”.

## Results

The presence of two bands (heavy chain and light chain) on SDS-PAGE gel (12.5%) indicated the purity of anti-rVP28 antibody (pAb) (S1 Fig). The titer of the purified antibody, as determined by ELISA was found to be 1:5000 (S2 Fig). Using ELISA, the LOD was observed to be 0.0078 μg/mL of purified WSSV protein (S3 Fig). Western blot result confirms that the purified antibodies do not cross-react with the shrimp tissue (S4 Fig).

**Fig 1:**
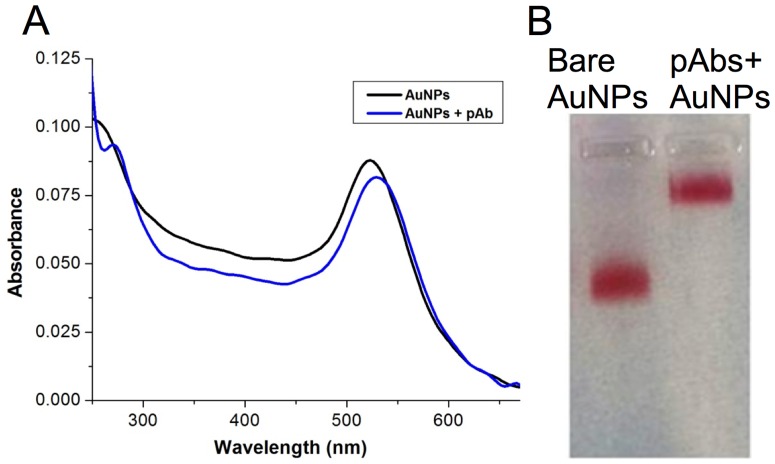
(A) UV-visible spectra of AuNPs and pAb-AuNPs, (B) Gel retardation assay of bare AuNPs and pAbs-AuNPs.

The as-synthesized AuNPs exhibited a characteristic surface plasmon resonance peak at 520 nm (with a high symmetry and narrow width), which exhibited a red shift upon addition of pAb confirming its conjugation with AuNPs (Fig 1A). Gel retardation assay also confirmed conjugation as evidenced by decreased mobility of pAb-AuNPs (Fig 1B) as compared to AuNPs alone. ELISA was performed using anti rVP28 –AuNPs conjugate as a label and the LOD was observed to be 0.0625 μg/mL of purified WSSV protein (S5 Fig).

**Fig 2:**
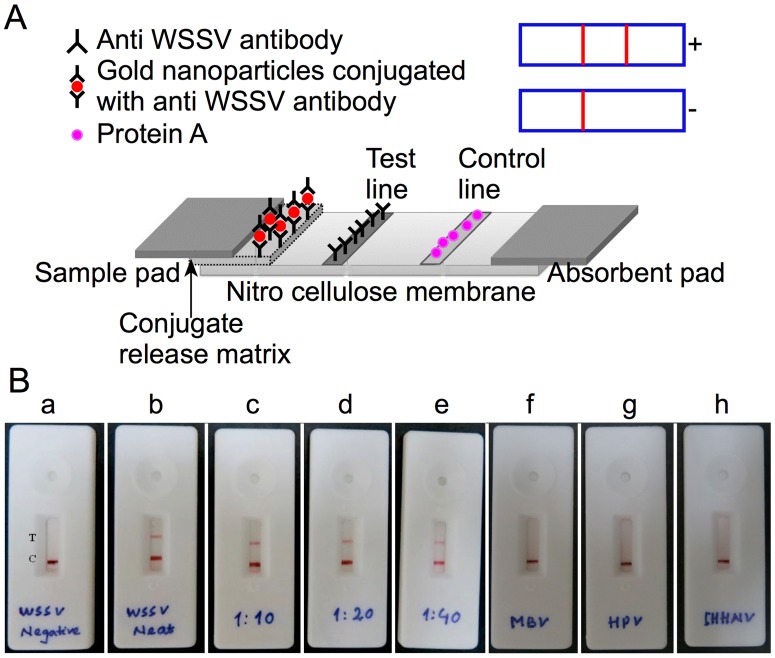
A. Schematic representations showing the LFIA strip assembly. B. Testing of WSSV in LFIA. (a) WSSV negative sample, (b) WSSV neat sample containing purified virus, (c), (d) and (e) correspond to 1:10, 1:20 and 1:40 dilutions of (b) Specificity of LFIA (f) MBV (g) HPV (h) IHHNV. C: Control line, T: Test Line.

**Fig 3:**
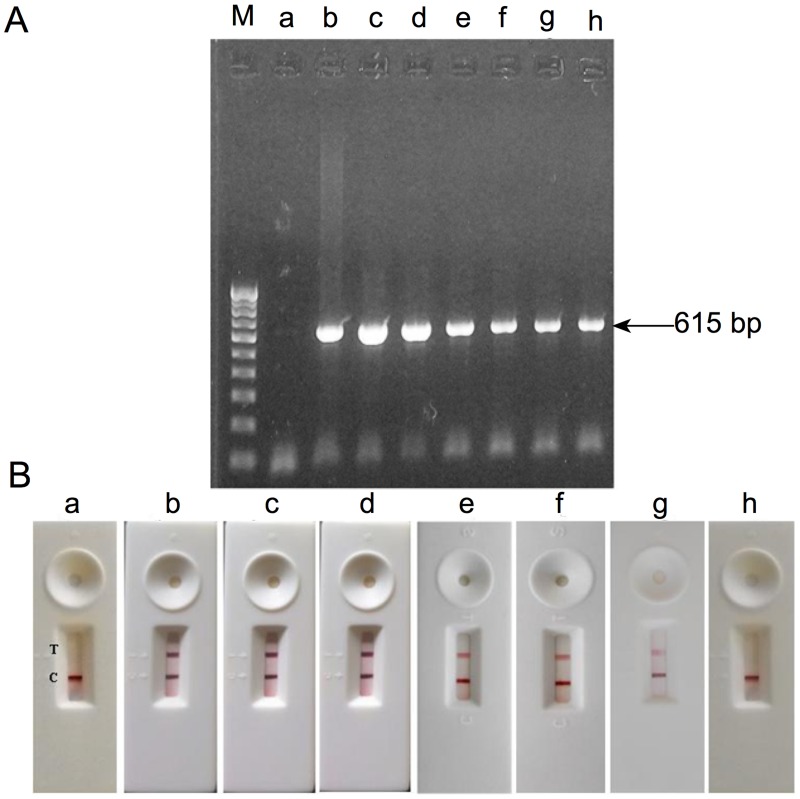
PCR of gill tissue sample from WSSV infected *Litopenaeus vannamei*. (A) M: DNA ladder. a: Uninfected gill tissue sample, b: WSSV infected gill sample (Positive control), c to h: Serially double diluted WSSV infected gill sample with concentrations from 100 to 3.125 μg/mL. (B) LFIAs of gill tissue sample from WSSV infected *Litopenaeus vannamei*. a:Uninfected gill sample b: WSSV infected gill sample (positive control), c to h: Serially double diluted WSSV infected gill tissue sample with a protein content of 100 to 3.125 μg/mL. From these 40 μL was applied in LFIA.

Assembly of the lateral flow immunoassay test strips is schematically represented in Fig 2A. To establish the workability of LFIA, purified WSSV samples were allowed to flow in the Nitrocellulose membrane. Red lines in the test and control zones could be visualized easily within 20 min. The presence of two red lines indicated a positive reaction confirming the presence of WSSV in the sample. The appearance of a red line only in control zone suggested the absence of WSSV, whereas formation of no line in the control and test zones indicated an invalid assay. Subsequently, the assay was carried out using WSSV samples that were subjected to two-fold dilutions (Fig 2B). To determine the specificity of the assay, the LFIA was further tested with other shrimp viruses such IHHNV, MBV, and HPV. The developed LFIA did not show any cross-reactivity with other shrimp viruses as evidenced by the appearance of a single red line only in the control zone of the LFIA (Fig 2B). The developed LFIA yielded a positive result up to protein concentration of 6.2 μg/mL when 40 μL of sample was applied (S6 Fig). The conventional one-step PCR could detect WSSV with template DNA isolated from the sample with a protein concentration of 3.125 μg/mL (Fig 3A and 3B).

**Fig 4:**
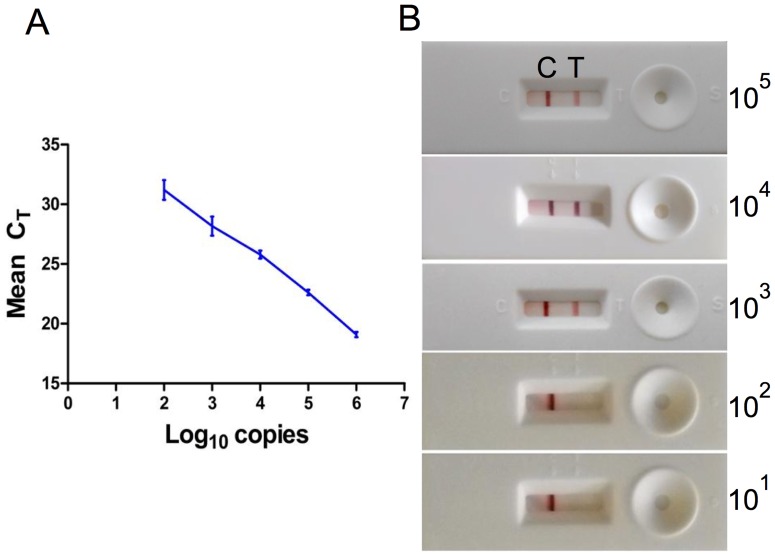
Standard curve for the real time PCR assay. (A) The virus was serially diluted (10-fold) from 2.4 x 10^7^ to 2.4 x 10^2^ copies prior to RT-PCR assay. (B) The LOD of LFIA, serially diluted samples were applied to LFIA. Control and Test line were observed visually.

Aliquots of WSSV infected gill samples, which were processed by LFIA were also used for DNA isolation and subjected to real time PCR using TaqMan probe to determine the limit of detection (LOD). Virus copy number in gill tissue homogenate was determined using a standard curve and was found to be 2.4 x10^7^ copies of WSSV. The graph of copy number versus mean C_T_ value obtained was linear (Fig 4A) and showed good correlation (y = -2.978x + 0.116; r^2^ = 0.9977). LFIA results (depicted in Fig 4B), clearly shows strong red lines for the sample containing 10^5^, 10^4^ and 10^3^ copies of WSSV. Thus, the limit of detection (LOD) of LFIA was found to be 10^3^ copies of WSSV.

### Time course infectivity experiment for early detection

During the early onset of infection, i.e. 3 h post-infection (p.i), hemolymph samples gave a positive readout in LFIA, whereas results were negative for all other tissue samples. With the progression of the disease, the LFIA was able to detect WSSV from hemolymph as well as gills of an infected animal at 6 h p.i. Subsequently, LFIA was able to detect the virus from head soft tissue, eye stalk, and pleopod at 12, 24, 36 h p.i. and the moribund stage. The results of LFIA were in complete agreement with one-step PCR (Table 1). The images indicating the results of LFIA and one step PCR are provided in S7 Fig.

**Table 1:** Time course infectivity experiments.

	Time of WSSV detection Post infection (h)											
	LFIA						One -step PCR					
**Organs**	**3**	**6**	**12**	**24**	**36**	**48**	**3**	**6**	**12**	**24**	**36**	**48**
**Hemolymph**	+	+	+	+	+	+	+	+	+	+	+	+
**Gill tissue**	**-**	+	+	+	+	+	**-**	+	+	+	+	+
**Head soft tissue**	**-**	**-**	+	+	+	+	**-**	**-**	+	+	+	+
**Eye stalk**	**-**	**-**	**-**	**-**	+	+	**-**	**-**	**-**	**-**	+	+
**Pleopod**	**-**	**-**	+	+	+	+	**-**	**-**	+	+	+	+

WSSV negative; + WSSV positive. Amount of protein applied in LFIA was 6.2 μg

### Validation of LFIA using field samples

Tissues of animals suspected of WSSV infection collected from farms (75) were analyzed simultaneously using one-step PCR and LFIA. In one-step PCR (conventional gold standard) 60% samples (45 out of 75) were diagnosed as positive or infected and the remaining 40% (30 out of 75) were found to be negative. When all the above samples were analyzed using LFIA, 44 samples tested positive and only one sample (which was positive in PCR) tested negative. Thus, the sensitivity and specificity of LFIA were 100 and 96.77%, respectively. The Pr (a) and Pr (e) values were found to be 0.9867 and 0.517, respectively while the value of Cohen’s kappa was calculated to be 0.972. The positive predictive value (PPV) and negative predictive value (NPV) obtained were 0.977 and 1, respectively (Table 2).

**Table 2:** Validation of LFIA.

		**One- step PCR**		
**LFIA**		Positive	Negative	**Total**
	Positive	44	1	**45**
	Negative	0	30	**30**
	**Total**	**44**	**31**	**75**
**Parameter**			**Result**	
Performance efficiency (%)			98.76	
Sensitivity (%)			100	
Specificity (%)			96.17	
FP rate (%)			3.23	
FN rate (%)			0	
Cohen’s Kappa coefficient			0.972	
Positive prediction value			0.977	
Negative prediction value			1	

## Discussion

Detection of WSSV is of enormous significance owing to severe economic losses incurred by the aquaculture industry throughout the world every year. In the absence of any therapeutic measures, prevention of infection is the only option available to check the spread of the disease. Currently, control methods aim at minimizing outbreaks of WSSV by reducing the stocking density, strict screening and monitoring of nauplii and post-larvae (PL) for infection. Apart from these control measures, accurate diagnosis of the virus at early stages of the disease is critical to minimize the losses. Hence, there is a need to detect the disease in a rapid, simple and field-usable manner, which was the primary focus of the current study.

The developed LFIA uses anti WSSV-rVP28 antibody coupled with AuNPs as the detection label. The spectrophotometric analysis of AuNPs before and after coupling with pAbs indicates a small shift in the absorbance peak from 520 nm to 522 nm. The observed shift in plasmon resonance peak suggested antibody coupling on gold nanoparticles. As reported by Ambrosi et al. 2010 [29] the interaction of AuNPs and the antibody could occur by three different phenomena, viz. (a) ionic interaction between the negatively charged gold nanoparticles and positively charged antibody molecule, (b) hydrophobic interaction between the antibody and the surface of gold nanoparticles (c) dative bonding between the conducting electron of colloidal gold and sulfur-containing amino acid of an antibody molecule. Furthermore, the appearance of an additional peak at 280 nm and the decrease in peak intensity at 520 nm confirms pAbs conjugation onto the surface of AuNPs.

As compared to one-step PCR, the developed LFIA was found to be marginally less sensitive. However, its ease of use under the field conditions without the requirement of any specialized instrument or trained workforce distinctly stands out. The LFIA developed in the present study can detect WSSV within 3 h p.i., much before the development of gross morphological signs of infection only consolidate its utility.

Our investigations and validation of the assay in the field prove that the specificity and sensitivity of the developed LFIA were comparable to one-step PCR, on the samples tested. The Cohen’s kappa coefficient (0.983) indicates “very good concordance” between the LFIA developed in the present study and conventional one-step PCR. A high negative predictive value indicates that the chances of getting a false negative result are remote in case of the developed LFIA.

Till date, many laboratory scale serological methods have been developed for the rapid and sensitive detection of viruses of shrimps and prawn. To the best of our knowledge, Shrimple test kit is the only commercialized product launched in the market. In a detailed study carried out evaluating the efficiency and sensitivity of Shrimple [16], it was seen that the results matched with the real-time PCR assay at 24 h post infection. During early onset of infection (1–8 h post-infection) all the samples tested negative in Shrimple assay. The lowest true positive results observed corresponded to 356 viral copies/ng of genomic DNA. The 50% true positive and equal number of false negative results as reported in the study [16] cast doubt on its reliability as an effective surveillance test.

The test strip developed for WSSV detection [17] had good specificity as evidenced by the fact that assay could not detect WSSV in un-infected *Penaeus monodon* or *Macrobrachium rosenbergii* or from Yellow head virus negative or Taura syndrome virus negative shrimps. However, it was less sensitive than dot blot method with the W29 monoclonal antibody. Therefore, this test described by Sithigorngul et al [17] was not recommended for screening of broodstocks showing least level of infection, post larvae used to stock shrimp ponds or other potential carriers for WSSV. Furthermore, the assay described by Sithigorngul et al [17] involves two monoclonal antibodies and one polyclonal antibody. The costs involved in the production of three antibodies would lead to the higher manufacturing cost of LFIA.

The LOD as calculated in the present study i.e. 1000 copies of WSSV is by far the lowest true chromatographic result using the polyclonal antibody for the detection of WSSV. The inherently high specificity of the rabbit polyclonal anti-rVP28 raised in rabbits stems from the use of a purified recombinant VP28. This, in turn, explains the observed high sensitivity. We believe that the high specificity polyclonal antibody obtained in the present study is akin to a monoclonal anti-VP28. Cross-reactivity studies with other shrimp viruses (MBV, HPV, and IHHNV) corroborate these findings. The observation assumes special importance because most of the overt symptoms of infection with other viruses are often overlapping and simultaneous infection with two or more viruses in a single cell type are not uncommon in shrimps [30].

The economic impact of widespread use of LFIA test could be considerable, both in terms of savings in the testing charges and preventing losses due to the control of infection. Usually, the shrimp farmers in countries like India have to screen all samples for WSSV by PCR test, which incurs an expenditure of ~USD 15 per sample (unpublished data collected by authors). LFIA (costing ~USD 3 per sample) could serve as the first-level screening test to eliminate WSSV positive samples. LFIA negative samples could then be tested by PCR, if required. Moreover, the farmer could perform the test without any outside professional help. Thus, the financial burden on the the farmer could be reduced substantially. The LFIA method developed here could be easily upgraded and translated into a multiplexed format for simultaneous detection of WSSV, IHHNV, MBV, HPV etc. In the absence of suitable prophylactic and treatment measures for viral diseases of shrimps, LFIA tests would directly benefit the farmers, hatchery operators and aquaculture industry and are destined to forestall heavy economic losses.

## Supporting Information

S1 Fig Purity of the IgG. T**o** check the purity of the purified IgG for rVP28 protein SDS-PAGE (12.5%) was run. Presence of heavy chain and light chain in the SDS-PAGE indicates the purity of IgG.
(TIFF)

S2 Fig Determination of titre for Anti rVP28-antibodies by ELISA. T**o** determine the titre of the anti-rVP28 antibodies an ELISA was performed. For this, 100 μg/mL of recombinant VP28 protein was coated onto the wells of high binding 96-well plates (Corning, USA) in triplicates and incubated overnight at 4°C. Wells were blocked with the blocking buffer (0.1 M NaHCO_3_ with 2 mg/mL bovine serum albumin, pH 8.6), washed thoroughly with PBS-T (PBS and 0.1% Tween-20, pH 7.4). Different dilutions of purified anti rVP28 antibody (1:500, 1:1000, 1:25000:1:5000, 1:7500 and 1:10000) were added and incubated for 1.5 h. After washing with phosphate buffer saline (PBS) containing tween-20(0.1%), HRP conjugated anti-rabbit secondary antibody was added in the wells and incubated for 1 h at RT. Post incubation, wells were washed with PBS-T, substrate (TMB-H_2_O_2_) was added and the incubated for 15 min. The reaction was terminated by adding 100 μL HCl (1M), and absorbance was monitored at 450 nm with the help of plate reader (Synergy Biotek, USA).
(TIFF)

S3 Fig Sensitivity of the anti rVP28 antibodies in ELISA. To determine the sensitivity of the ELISA using anti rVP28 antibodies, the assay was carried out using different concentrations of purified virus, viz., 0.0039 to 2 μg/mL. Following blocking with 0.1 M NaHCO_3_ with 2 mg/mL bovine serum albumin, pH 8.6, wells were washed thoroughly PBS-T (PBS and 0.1% Tween-20, pH 7.4). Post washing, 1:5000 dilution of purified anti-rVP28 antibody was added and incubated for 1.5 h. After washing with Phosphate buffer saline (PBS) containing tween-20(0.1%), HRP conjugated anti rabbit secondary antibody was added in the wells and incubated for 1 h at RT. Post incubation, wells were washed with PBS-T, substrate (TMB-H_2_O_2_) was added and the incubated for 15 min.
(TIF)

S4 Fig Western blot using anti WSSV antibodies. Initially, gill and pleopod t homogenates of WSSV infected *Litopenaeus vannamei* and tissue homogenate of *Litopenaeus vannamei* without any infection were separated on 12.5% SDS-PAGE and transferred on to a polyvinylidene difluoride (PVDF) membrane. The membranes were blocked at ambient temperature (25±3°C) in blocking buffer (5% skimmed milk in TBS and 1% Tween-20) for 2 h. Post incubation, the membranes washed for 10 min with TBST and then incubated with HRP conjugated secondary antibody (1:5000 dilutions) at 37°C for 2 h. The signals on the membrane were developed by diamino benzidine (DAB, Sigma, USA) as substrate. Fig_S4 indicates the western blot analysis for detecting WSSV using antiserum raised against recombinant VP28 protein A: pre-stained marker B: tissue homogenate of healthy shrimp tissue C: *Gill tissue homogenate of Litopenaeus vannamei* infected with WSSV D: *Pleopod tissue homogenate of Litopenaeus vannamei*.
(TIFF)

S5 Fig ELISA using anti rVP28 –AuNPs. To determine the sensitivity of the ELISA using anti rVP28 antibodies, the assay was carried out using different concentrations of purified virus, viz., and 0.03125 to 4 μg/mL. Following blocking with 0.1 M NaHCO_3_ with 2 mg/mL bovine serum albumin, pH 8.6, wells were washed thoroughly PBS-T (PBS and 0.1% Tween-20, pH 7.4). Then the diluted rVP28-AuNPs (100 μL) was added to each well and incubated for 1 h. Post incubation, the wells were washed for three times with PBST. The absorbance was measured at 520 nm.
(TIFF)

S6 Fig Intensity plot of LFIA. After performing the LFIA with different concentration of WSSV the intensity of the control line and test line was measure by Image J software. The intensity was calculated with average intensity of the test line divided by sum of the intensity of the control and test lines in each strip. The intensity was plotted against different antigen concentrations. The data were fitted linearly.
(TIF)

S7 Fig Images indicating early detection by LFIA and one step PCR is shown in this figure. (TIFF)
