# Supplementary figures and images for: Field-Usable Lateral Flow Immunoassay for the Rapid Detection of White Spot Syndrome Virus (WSSV)
Source: PLoS One. 2017 Jan 3;12(1):e0169012. doi: 10.1371/journal.pone.0169012 (PMC5207695; doi:10.1371/journal.pone.0169012)

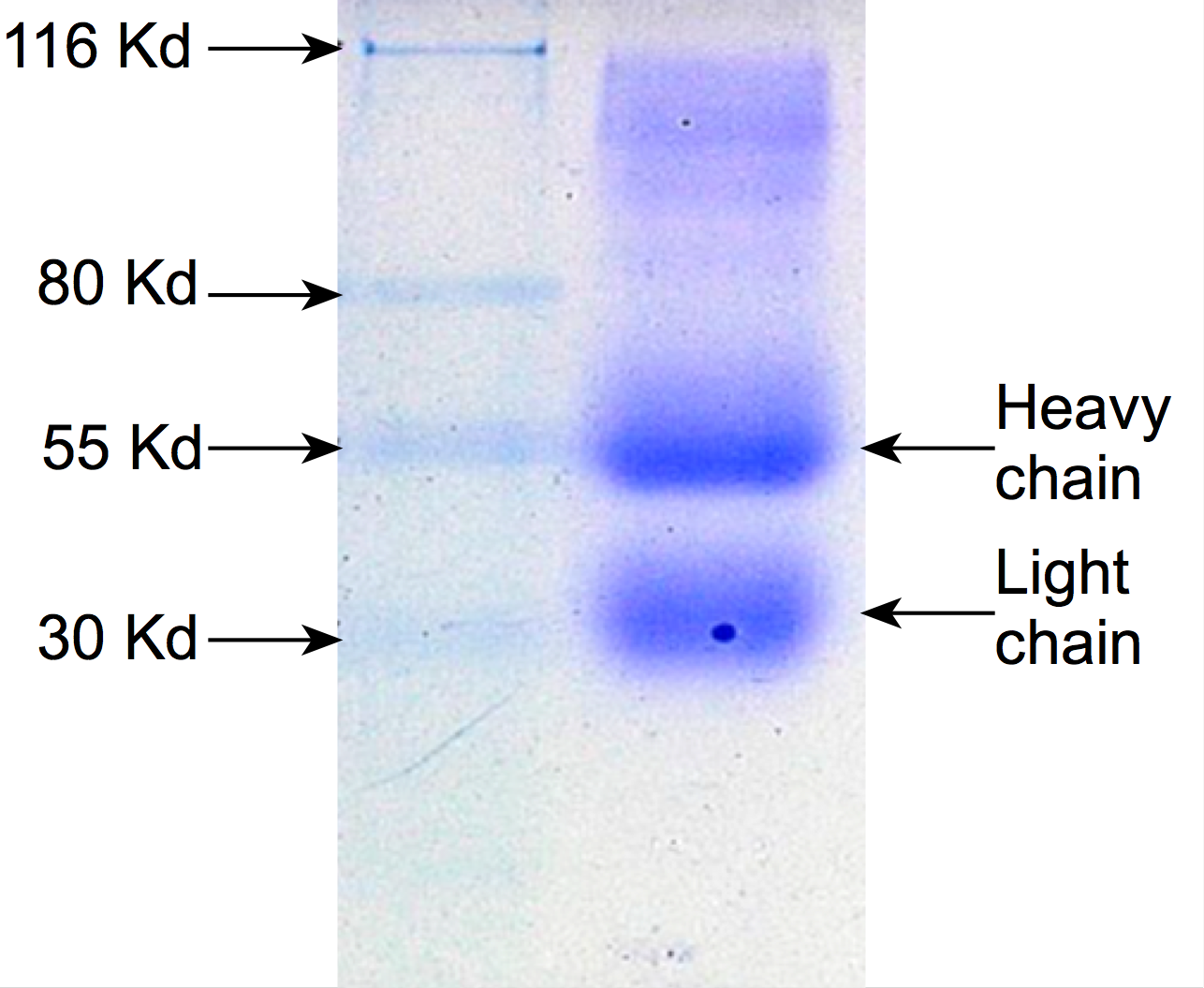

Supplement: S1 Fig — To check the purity of the purified IgG for rVP28 protein SDS-PAGE (12.5%) was run. Presence of heavy chain and light chain in the SDS-PAGE indicates the purity of IgG. (TIFF) [file pone.0169012.s001.tiff]

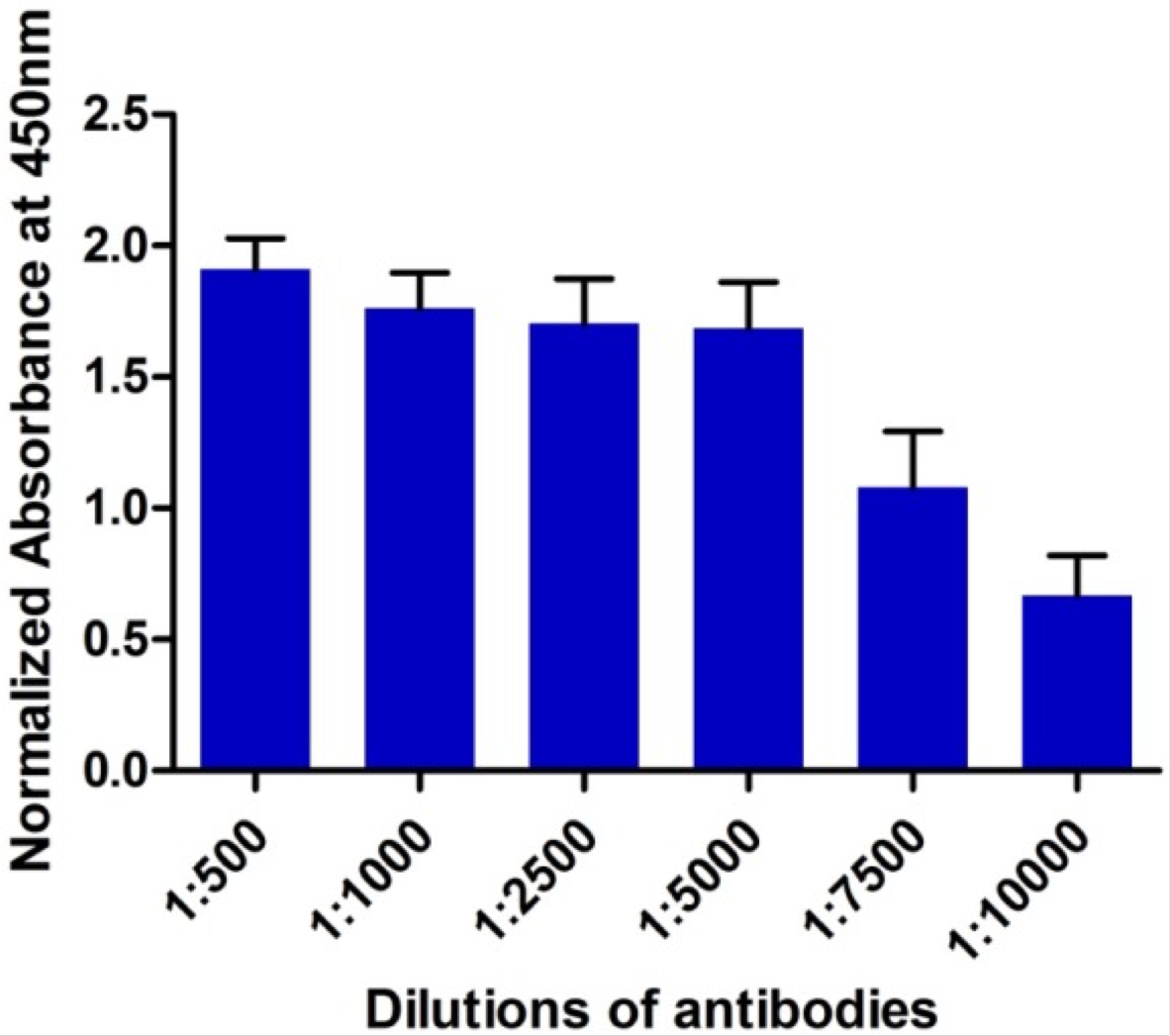

Supplement: S2 Fig — To determine the titre of the anti-rVP28 antibodies an ELISA was performed. For this, 100 μg/mL of recombinant VP28 protein was coated onto the wells of high binding 96-well plates (Corning, USA) in triplicates and incubated overnight at 4°C. Wells were blocked with the blocking buffer (0.1 M NaHCO3 with 2 mg/mL bovine serum albumin, pH 8.6), washed thoroughly with PBS-T (PBS and 0.1% Tween-20, pH 7.4). Different dilutions of purified anti rVP28 antibody (1:500, 1:1000, 1:25000:1:5000, 1:7500 and 1:10000) were added and incubated for 1.5 h. After washing with phosphate buffer saline (PBS) containing tween-20(0.1%), HRP conjugated anti-rabbit secondary antibody was added in the wells and incubated for 1 h at RT. Post incubation, wells were washed with PBS-T, substrate (TMB-H2O2) was added and the incubated for 15 min. The reaction was terminated by adding 100 μL HCl (1M), and absorbance was monitored at 450 nm with the help of plate reader (Synergy Biotek, USA). (TIFF) [file pone.0169012.s002.tiff]

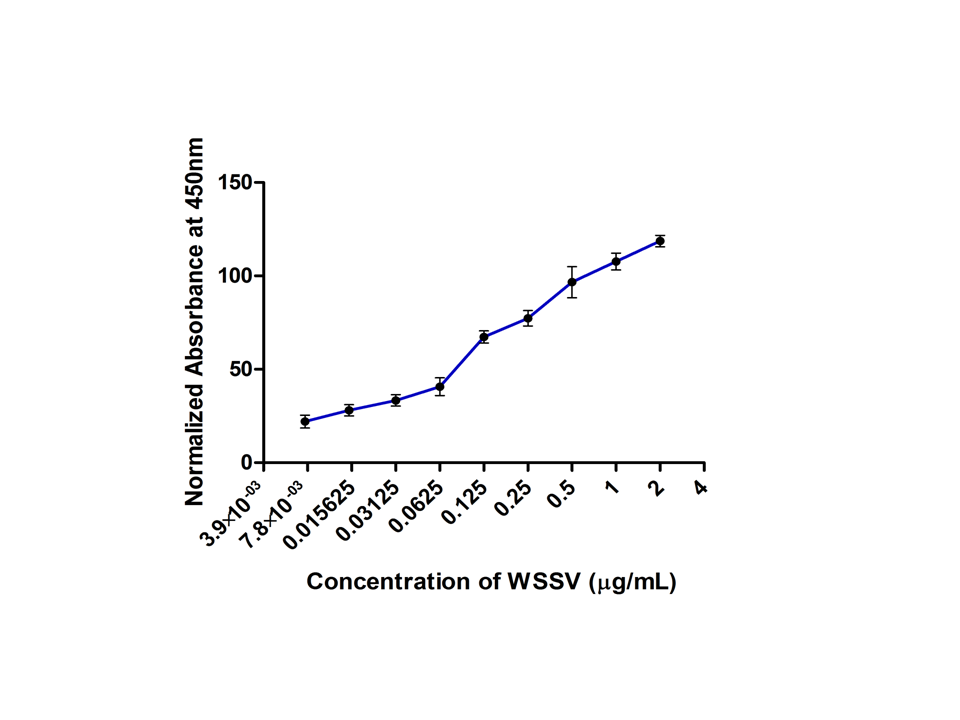

Supplement: S3 Fig — To determine the sensitivity of the ELISA using anti rVP28 antibodies, the assay was carried out using different concentrations of purified virus, viz., 0.0039 to 2 μg/mL. Following blocking with 0.1 M NaHCO3 with 2 mg/mL bovine serum albumin, pH 8.6, wells were washed thoroughly PBS-T (PBS and 0.1% Tween-20, pH 7.4). Post washing, 1:5000 dilution of purified anti-rVP28 antibody was added and incubated for 1.5 h. After washing with Phosphate buffer saline (PBS) containing tween-20(0.1%), HRP conjugated anti rabbit secondary antibody was added in the wells and incubated for 1 h at RT. Post incubation, wells were washed with PBS-T, substrate (TMB-H2O2) was added and the incubated for 15 min. (TIF) [file pone.0169012.s003.tif]

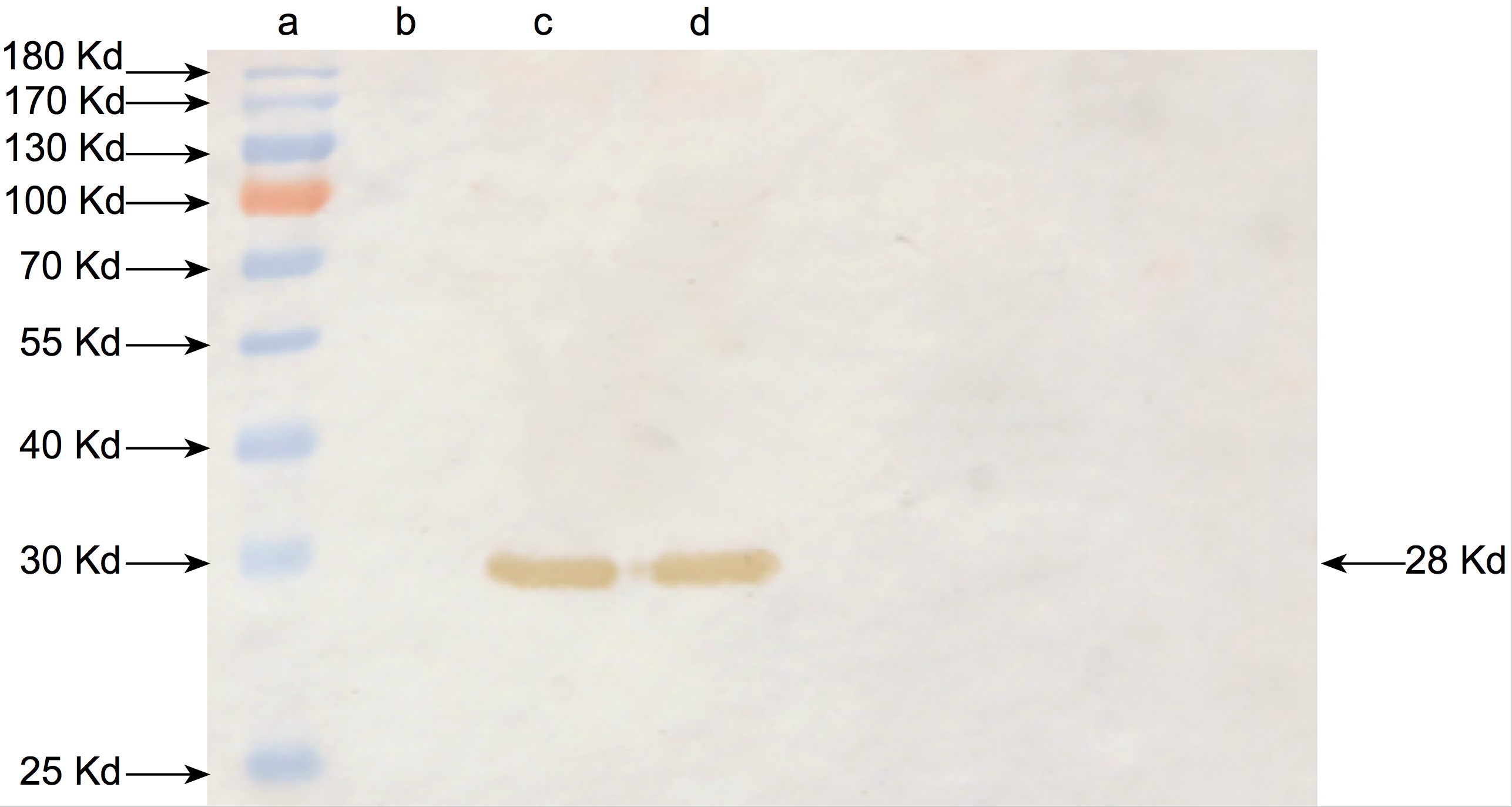

Supplement: S4 Fig — Initially, gill and pleopod t homogenates of WSSV infected Litopenaeus vannamei and tissue homogenate of Litopenaeus vannamei without any infection were separated on 12.5% SDS-PAGE and transferred on to a polyvinylidene difluoride (PVDF) membrane. The membranes were blocked at ambient temperature (25±3°C) in blocking buffer (5% skimmed milk in TBS and 1% Tween-20) for 2 h. Post incubation, the membranes washed for 10 min with TBST and then incubated with HRP conjugated secondary antibody (1:5000 dilutions) at 37°C for 2 h. The signals on the membrane were developed by diamino benzidine (DAB, Sigma, USA) as substrate. Fig_S4 indicates the western blot analysis for detecting WSSV using antiserum raised against recombinant VP28 protein A: pre-stained marker B: tissue homogenate of healthy shrimp tissue C: Gill tissue homogenate of Litopenaeus vannamei infected with WSSV D: Pleopod tissue homogenate of Litopenaeus vannamei. (TIFF) [file pone.0169012.s004.tiff]

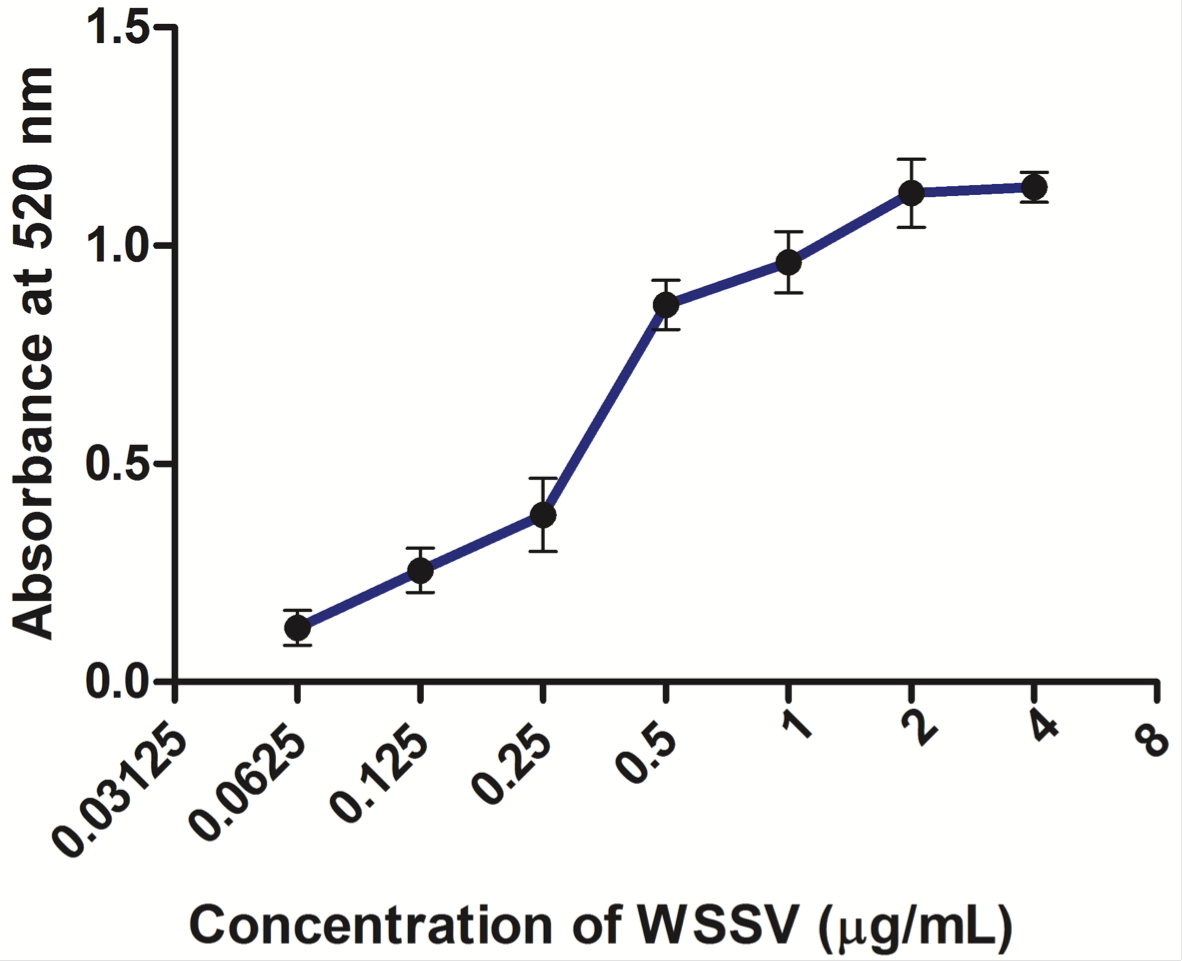

Supplement: S5 Fig — To determine the sensitivity of the ELISA using anti rVP28 antibodies, the assay was carried out using different concentrations of purified virus, viz., and 0.03125 to 4 μg/mL. Following blocking with 0.1 M NaHCO3 with 2 mg/mL bovine serum albumin, pH 8.6, wells were washed thoroughly PBS-T (PBS and 0.1% Tween-20, pH 7.4). Then the diluted rVP28-AuNPs (100 μL) was added to each well and incubated for 1 h. Post incubation, the wells were washed for three times with PBST. The absorbance was measured at 520 nm. (TIFF) [file pone.0169012.s005.tiff]

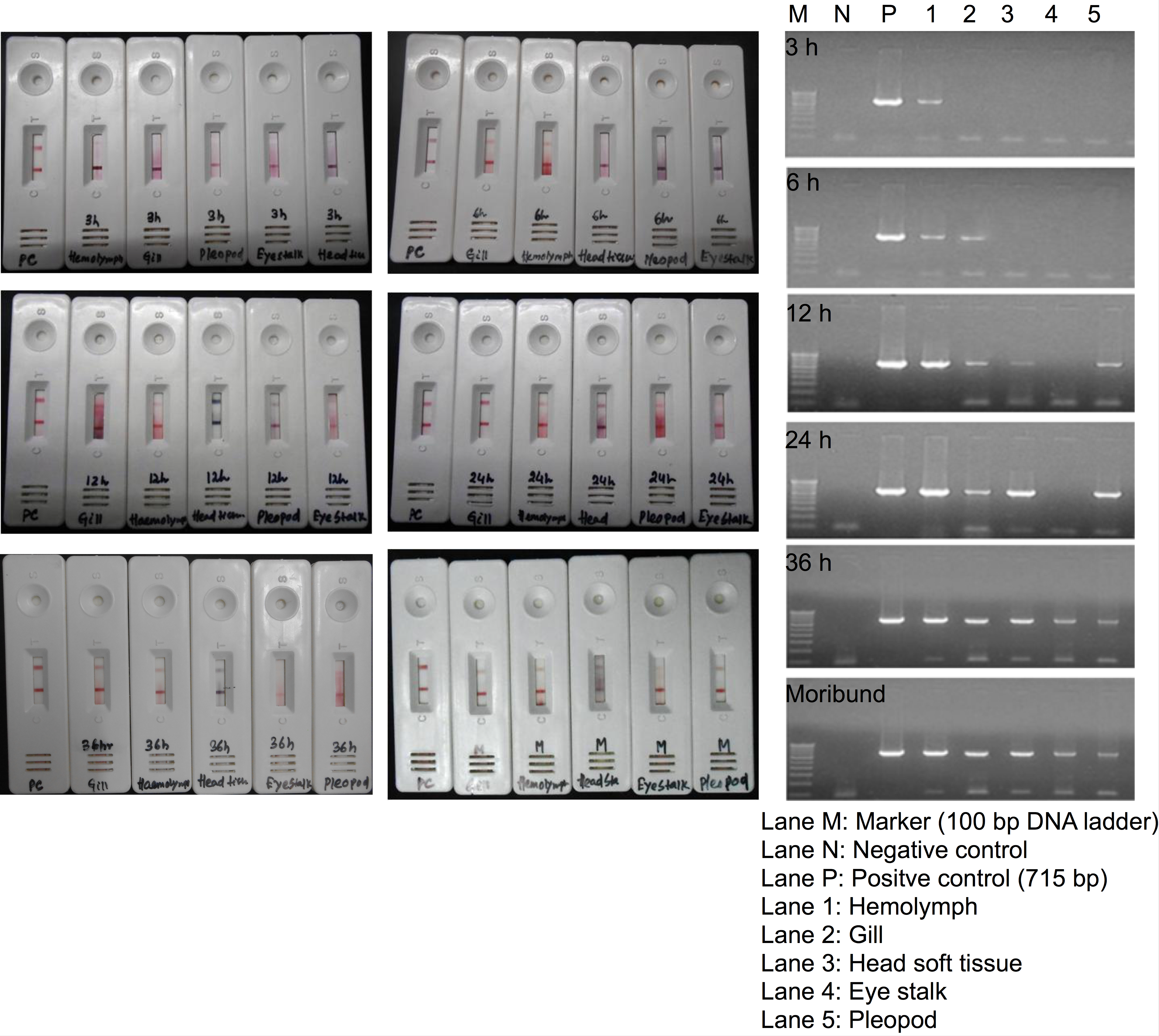

Supplement: S7 Fig — (TIFF) [file pone.0169012.s007.tiff]
